# Habitat degradation and predators have independent trait-mediated effects on prey

**DOI:** 10.1038/s41598-019-51798-2

**Published:** 2019-10-31

**Authors:** Mark I. McCormick, Eric P. Fakan, Maria M. Palacios

**Affiliations:** 1 ARC Centre of Excellence for Coral Reef Studies, and Department of Marine Biology and Aquaculture, James Cook University, Townsville, Queensland 4811 Australia; 2 Present Address: Centre for Integrative Ecology, Deakin University, Melbourne, Victoria 3125 Australia

**Keywords:** Behavioural ecology, Climate-change ecology

## Abstract

Coral reefs are degrading globally leading to a catastrophic loss of biodiversity. While shifts in the species composition of communities have been well documented associated with habitat change, the mechanisms that underlie change are often poorly understood. Our study experimentally examines the effects of coral degradation on the trait-mediated effects of predators on the morphology, behaviour and performance of a juvenile coral reef fish. Juvenile damselfish were exposed to predators or controls (omnivore or nothing) in seawater that had flowed over either live or dead-degraded coral over a 45d period. No interaction between water source and predator exposure was found. However, fish exposed to degraded water had larger false eyespots relative to the size of their true eyes, and were more active, both of which may lead to a survival advantage. Non-consumptive effects of predators on prey occurred regardless of water source and included longer and deeper bodies, large false eyespots that may distract predator strikes away from the vulnerable head region, and shorter latencies in their response to a simulated predator strike. Research underscores that phenotypic plasticity may assist fishes in coping with habitat degradation and promote greater resilience to habitat change than may otherwise be predicted.

## Introduction

Habitat degradation is one of the key drivers of a global catastrophic loss of biodiversity^1–3^. The loss and modification of ecosystem engineers, such as large canopy trees or rapidly growing hard corals, impacts communities through changes in the diversity of the landscape and services it provides^4^. Because habitat degradation is usually a gradual process, its impact on communities is first detected through changes in the physiology of species^5^. Physiological challenges sum to whole-animal changes in body condition by affecting an individual’s overall health and ability to obtain limited resources^6,7^. The way an individual changes physiologically, and how these changes alter interactions within a community, determines an individual’s fitness and the resilience of the species to habitat degradation^8^. While shifts in the species composition of communities have been well documented associated with habitat change (e.g.^9,10^), the mechanisms that underlie change are often poorly understood.

Changes to the composition of landscape-forming species alters the sensory environment of the animals that live within the habitat^11,12^. The interactions between individuals that make up community dynamics are framed within a foundation of current and historical information from their local environment^13,14^. The information within which to frame decisions changes as the availability of sensory information changes. For instance, a loss of a forest canopy alters the visibility and passage of air through the environment, altering the information available from visual and olfactory senses^15^. This change in the availability of fundamental information can alter the outcome of interactions between individuals and species, which are the basis of community dynamics. Predator-prey interactions have evolved to be information-demanding, because predators have usually coevolved with their prey, making both predator attack and prey escape strategies highly efficient^16^, Alterations to the flow and types of information through the community are therefore very likely to affect predator-prey dynamics and may be one of the fundamental mechanisms that underlie the rapid change in community composition that comes with habitat change.

Prey have evolved to be highly responsive to the threat of predation, and predation is viewed as the driving force underlying physiology, morphology and behaviour through selective loss. Not only do predators have direct (lethal or consumptive) effects on prey, but they also have indirect (non-consumptive), trait-mediated effects on prey through the threat of predation. Indeed, meta-analyses find that these non-consumptive ‘fear-effects’ have more of an impact on prey ecology, and consequently community dynamics, than their consumptive effects alone. This has been well demonstrated in terrestrial^17^, freshwater^18^ and marine systems^19,20^. Studies of aquatic organisms have found that the sight or smell of a predator can lead to changes in life history traits (e.g., developmental rates and hatching times^21^;), behaviour (e.g., development of neophobic phenotypes^22^;) and alter body morphology^23^ to forms that are best suited to surviving in a location where the threat of predation is high. Landmark examples of this are the deepening of the body in crucian carp (*Carassius carassius*) to the smell of predatory pike (*Esox lucius*)^24^, and the increase in the relative size of the false eyespot in damselfish (*Pomacentrus amboinensis*) prey, believed to attract predator strikes to the less vulnerable tail region^23^. Developmental plasticity in these morphological characteristics has been shown to affect performance and survival^23,25,26^, underscoring the importance of trait-mediated effects in prey ecology and fitness.

Many aspects of predator-prey interactions in aquatic environments are mediated by olfactory cues and changes in the chemistry of the surrounding environment can have impacts on the efficacy of cues^27^. Learning and updating information about predators principally occurs for many aquatic organisms through chemical associative learning, whereby damage-released chemical cues (known as alarm odours) label other unrelated stimuli (e.g., sight, sound or smell of a predator) as a threat when they co-occur^28^. Recent research has shown that many fishes lose their ability to use alarm odours in degraded coral reef habitats due to changes in the background chemistry of the environment, which directly impacts survival in the field^29^. While studies have shown that prey survival is compromised in degraded habitats due to uninformed decisions^30,31^, it is unknown whether the degradation of habitats such as coral reefs alters the action of trait-mediated antipredator solutions.

The current study examines the effects of habitat degradation on trait-mediated antipredator traits in a coral reef fish. We focus on the coral reef ecosystem as it is one of the most biodiverse on the planet and because it is subject to degradation on a global scale, changing from hard-coral to algal dominated landscapes^9^. Previous studies have highlighted the importance for prey of olfactory information on the activity of predators^28^, and the smell of predators alone have been previously shown to alter prey behaviour and morphology in water from healthy coral reefs^19,23,25^. The current study examines how water from healthy and dead-degraded habitats can influence the development of body morphology and escape performance in a habitat generalist damselfish, *Pomacentrus amboinensis*. This is a particularly interesting model species as it has a false eyespot (i.e., ocellus) as a juvenile whose size has been found to correlate with survival^23^. Predictions from previous research suggest water source will interact with the type of stimulus fish to which focal prey fish were exposed (i.e., blank control, herbivore or predator), because degraded water has been found to prevent the labelling of the predator as a threat^32,33^. Consequently, we expect that predator-associated trait-mediated effects, such as changes to body depth and the size of the false eyespot, would be negligible in degraded habitats compared to fish reared in water that has flowed over live coral.

## Materials and Methods

### Study system and species

The study was conducted at the Lizard Island research station (14°40'43.07"S, 145°26'53.35"E), on the northern Great Barrier Reef, Australia from October to December 2017. Newly metamorphosed damselfish (family Pomacentridae) were collected from light traps that had been deployed overnight at least 30 m from the reef edge and over a water column 8–20 m deep. The focal damselfish species, *Pomacentrus amboinensis*, is a common component of Indo-Pacific coral reefs where it is an omnivorous planktivore^34^.

The predator species used in the experiment was the dusky dottyback, *Pseudochromis fuscus* (family: Pseudochromidae), which is a common mid-sized predator on the shallow reefs throughout the Indo-Pacific. The species is a voracious predator of recently settled fishes during the recruitment season^35^ and is common in the same habitats used by *P. amboinensis*. The omnivorous goby, *Amblygobius phalaena*^36^, was used as the stimulus fish in experimental controls to account for the effect of exposing *P. amboinensis* to visual and olfactory cues of any heterospecific fish species. It is similar in body shape to the predator, *Ps. fuscus*, and is common on the base of the reef where *P. amboinensis* occurs^34^. Both species were caught by SCUBA divers using a small hand net and a dilute clove oil solution that anaesthetizes the fish and facilitates their capture from the reef.

Live bushy hard coral (*Pocillopora damicornis*) and dead-degraded coral covered in a mixture of algae and sessile invertebrates (450–500 ml in volume; dimensions ~200 × 150 × 150 mm) were collected from fringing reefs around Lizard Island, and placed in aerated 60 L header tanks of flow-through seawater^37^. Live and dead *Po. damicornis* are common nursery habitats for newly recruited damselfish, and commonly found within *Ps. fuscus* territories^35^.

### Experimental design and protocol

To examine the effect of water source on a prey’s morphological responses to a predation threat, individual *P. amboinensis* were exposed to six experimental treatments combining olfactory and visual cues of a predator (the dottyback, *Ps. fuscus*), a non-predator (the herbivorous goby, *A. phalaena*), or a blank control (receiving no cue from adult fish sources) in either seawater that had flowed over live coral or over dead-degraded coral, in a 3 × 2 design. Given that degraded water sources can limit the labelling of predators as threat^31–33^, we expected *P. amboinensis* from this treatment would not develop trait-mediated responses towards the dottyback and be morphologically similar to prey exposed to the control or herbivore fish.

Prior to being randomly allocated to one of the 6 treatment combinations, light trap caught *P. amboinensis* individuals were conditioned to recognize the sight and olfactory cues of *P. fuscus* in either water from live coral or dead-degraded coral. Conditioning was undertaken by chemical associative learning, using a commonly used protocol. Conspecific chemical alarm odours from five light trap caught *P. amboinensis* were added to 15 ml of treatment water (from live or dead-degraded coral header tanks) and 500 ml seawater from a 35 L tank that had 4 *Ps. fuscus* sitting under aeration, but no water flow for 1 hour. Alarm odours were collected from fish killed by cold shock (see animal welfare below) and superficially lacerated with five vertical cuts per side from a scalpel^38^. *Ps. fuscus* were also added to the tanks within a plastic bag filled with aerated seawater so that *P. amboinensis* were trained to associate the sight of a dottyback as a threat. This training procedure has been found to substantially increase the probability of survival in the Ambon damselfish^39^, and is necessary to make sure that prey can recognise the cues of the predator species within the appropriate environmental context into which they settled. Fish were specifically trained in the water treatment where they were going to be reared and tested, in order to maintain a realistic and consistent scenario throughout this study, as well as to capture any effect that the water treatment has on the ability of *P. amboinensis* to recognise predators^32,33^. This approach ensured that all fish within a water type (live coral vs. degraded coral) had the same baseline predator experience before the commencement of the study.

Trained damselfish (N = 144) were transferred to purpose-built experimental predator–prey tanks ( 52 L; see Supplementary Fig. S1). Each experimental tank consisted of four 6.1 L sections for the stimulus species (i.e., dottyback, goby or blank control) and 12 individual prey compartments (2.3 L). The design of the experimental tank allowed each of the stimulus compartments to exchange visual and chemical cues only with the three prey compartments in front (see Supplementary Material). The experimental tank included a 1.5 cm layer of sand on the bottom, PVC tubes providing shelter to the stimulus species and the prey, and a plastic branching coral skeleton (*Pocillopora* sp. ~5 × 3 × 8 cm) at the back of each *P. amboinensis* compartment to provide topography. Experimental tanks were situated in an outdoor wet laboratory to ensure that all fish received natural temporal cues and the seawater was supplied via header tanks containing either live coral or dead-degraded coral (as described above). *P. amboinensis* were individually fed twice daily with 10 ml of *Artemia* sp. nauplii (mean 1245 *Artemia*/ml), while dottyback predators were fed two euthanized *P. amboinensis* recruits at the start and end of the day. Gobies were fed 5 ml of small crustaceans from the light trap catches at the same time of day as the predators. A small number of fish were lost in the first 2 d after the initiation of the experiment (17 out of 144 due to plumbing issues; representing 11 fish from live and 6 fish within dead coral water sources). These were restocked with fish that had been conditioned together with the original fish used to stock the tanks.

Water from the Lizard Island research station seawater system was used for this study. We expect that the chemistry of the seawater fed into the system that formed the background water source for all treatments represented a mixture of healthy and degraded coral odours, given that the seawater is sourced from the island’s shallow backreef. Seawater from the station was passed through a one micron filter bag before entering the 60 L header tanks containing either the live *Po. damicornis* or the dead-degraded corals as above. A pipe on the opposite side of the header tank, allowed water to flow into the central chamber of the experimental tank that contained the stimulus species (i.e., dottyback, goby or nothing). Water then flowed through six holes in the Perspex divider to each of the compartments containing individual *P. amboinensis* and exited through holes in the back of their compartments. Dye trials confirmed this flow pattern. Given the arrangement of the tanks, water flow, and panels, each *P. amboinensis* received visual cues from the stimulus species and chemical cues from a combination of the water source (live or degraded coral) and the stimulus (dottyback, goby or blank control). *P. amboinensis* could neither see nor smell conspecifics in the nearby chambers due to the design of the tanks and the direction of the water flow.

Stimulus fishes (i.e., *Ps. fuscus* or *A. phalaena*) were replaced every 2 weeks, and specific individuals rotated every 2 days within treatments. Coral substrata were kept in the aquarium system for a maximum of one week prior to being replaced with newly collected material. In this way, each *P. amboinensis* was exposed to 42 days of continuous treatment, but the specific identity of the stimulus fishes and coral was frequently shifted within treatment. *P. amboinensis* were returned to their original compartments after being periodically photographed.

### Morphometric measures

*P. amboinensis* were individually placed inside a clip-seal bag containing aerated seawater (either live or degraded) and photographed on their side against a centimeter grid at the start (day one), midway through (day 22) and at the end (day 45) of the experiment. Using the software ImageJ, a variety of morphometric measures were taken from the images: (i) standard length (SL), (ii) body depth, (iii) area of the eye, (iv) area of the black portion of the eye, (v) area of ocellus, and (vi) area of the black portion of the ocellus (Fig. S2). At the end of the study, fish were weighed by transferring fish onto tissue paper prior to placing them in a tared quantity of seawater. Upon recovery from stress fish were released onto the fringing reef.

### Behavioural assessments

Behavioural assessments were conducted at the start (day 5), midway through (day 23) and at the end of the experiment (day 42). The procedure involved suspending a mirror (80 × 40 cm) over each tank at 45° so that focal fish could be filmed from above with a video camera positioned parallel to the tank and pointing at the mirror. The camera stood on a tripod and was always out of sight of the focal fish. After a 10 min acclimation period, individual *P. amboinensis* were fed 10 ml of *Artemia* sp. nauplii (mean 1245 *Artemia*/ml), while the video camera recorded their behaviour for 5 minutes at 30 fps. Similar to a previous study by Lönnstedt and colleagues^23^, the total number of feeding strikes and activity (i.e., number of seconds fish were not in shelter) were quantified from the first 120 s after the feeding event.

### Routine swimming

After 45 days individual *P. amboinensis* were removed from their compartments and exposed to a repeatable drop stimulus using the methodology of McCormick and Allan^40^. The testing arena consisted of a transparent circular acrylic area (diameter 200 mm; height 70 mm), within a large opaque-sided plastic tank (585 × 420 × 330 mm; 60 L) with a transparent Perspex bottom to allow responses to be filmed from below as a silhouette. The water level was low (60 mm) to reduce movements in the vertical plane and the arena was lit by an LED light strip attached to the outer surface of the opaque tank. Fish were not fed overnight (~16 h) and the water within the arena matched the water within the holding tanks of the tested fish. Fish were placed in a 1-litre opaque tank of matching water within a water bath in a quiet area for 30–40 minutes prior to being carefully transferred into the test arena.

After a five minute acclimation period within the arena, fish were videoed at 30 fps for two minutes to obtain an estimate of their activity. Activity measured as the total distance moved over the 120 s period measured at one second intervals.

### Escape performance assessment

Once routine swimming had been recorded, fish were exposed to a repeatable startle stimulus which followed the protocol of McCormick and Allan^40^. Fish were only startled when they moved to the middle portion of the tank and were oriented toward the drop stimulus, which is located in the center of the arena, allowing an individual to move an equal distance in any direction and standardising for fish position relative to the stimulus. A weight was released from an electromagnet and was governed by a piece of fishing line that was long enough such that the tapered tip only just touched the surface of the water. To avoid the occurrence of a premature escape response associated with visual stimulation, the weight was released from above through a 550 mm piece of 48.5 mm diameter PVC pipe with the bottom edge at a distance of 10 mm above the water level. Escape variables were only measured when prey performed a C-start (i.e., commencement of fast-start that results in the individual forming a C-shape). Escape responses were recorded at 480 frames per second (Casio EX-ZR1000) as a silhouette from below.

Kinematic variables associated with the fast-start response were analysed using the image-analysis software Image-J, with a manual tracking plug-in following standard protocols^40^. For analysis of the high-speed movements each fish was reduced to a single moving point. The point where each fish was tracked was standardised by following the same point on each fish (i.e., the position directly behind the eyes which corresponds to the widest part of the body). We chose to standardise tracking based on this point of the body as it the most stable and easiest to track owing to the small size of the juveniles. Distance to the drop stimulus was measured at the point of contact of the weight with the water surface. The following fast-start variables were measured^40^:

1. Response latency (ms) was measured as the time interval between the stimulus touching the water surface and the first detectable movement of the fish.
2. Response speed (m/s) was measured as the distance covered within a fixed time (24 ms). This fixed duration was based on the average duration (22.8 ms) of stage one and two (as defined above).
3. Maximum response speed (m/s) was measured as the maximum speed achieved at any time during stage one and stage two.

### Ethics statement

This research was undertaken in accordance with the animal ethics guidelines and regulations of the James Cook University, and all protocols were approved by the James Cook University Animal Ethics Committee (Approval Numbers: A2408). Animal welfare issues are detailed in the Supplementary File.

### Statistical analyses

Standard length (SL) was tested between water sources (Live coral, Dead-degraded coral), treatments (Control, Goby, Dottyback) and their interaction separately for the start and end of the experiment using general linear models (GLMs). Body weight was only measured at the end of the experiment, so had a more abbreviated analysis. Other body variables (e.g., body depth, max eye diameter) that were correlated with standard length were tested for effects of water source, treatments and their interaction using analysis of covariance, using SL as a covariate. A test of the assumption of homogeneity of slopes were run prior to running the reduced model. SL was not a significant covariate for tests on the proportion of ocellus to eye maximum diameter.

The routine swimming variable total distance moved was initially analysed with the fixed factors water source and treatment and SL as a covariate. SL and its interactions accounted for no significant variation so were dropped from the final analyses. Fast-start latency was correlated with distance to the drop stimulus, while mean speed and maximum speed had positive correlations (covariance) with SL, so these correlated variables were used as covariates in analyses. Because of the interdependence of kinematic variables an α’ = 0.0125 was used as the cut-off for interpretation to reduce the impact of type I error inflation.

All analyses were originally run as linear mixed effects models accounting for the tank effect, but this term was found to be of negligible importance (variance was usually <30% of the residual) and subsequently dropped from the analyses. For all analyses, assumptions of normality and homogeneity of variance were examined using residual analyses. Due to minor differences in sample sizes among treatment by water source combinations (n = ~24, though a small number died; for exact N see figure legends) Type III sums of squares were used in GLM analyses. Effect sizes are given as partial-eta-squared, which represents the proportion of the total variance in a dependent variable that is associated with the membership of different groups.

## Results

### Morphology

At the start of the experiment, there was a significant difference in the length of damselfish between water sources (F_1,125_ = 5.78, P = 0.02, η_p_^2^ = 0.04), with fish in the live coral water treatment being slightly longer than those exposed to dead-degraded seawater (mean 12.0, 11.8 mm SL respectively, SE 0.07). This was likely due to the restocking of fish that were lost at the start of the experiment within the treatments (11 live vs 6 dead coral treatments). There was no difference in size-corrected body depth among treatments, water sources or their combination at the start of the experiment (P > 0.2). Initial trends in means size were very different from their patterns at the end of the experiment (below), suggesting that the small initial differences (i.e., 0.2 mm in length) had little effect on the final patterns found.

**Figure 1:**
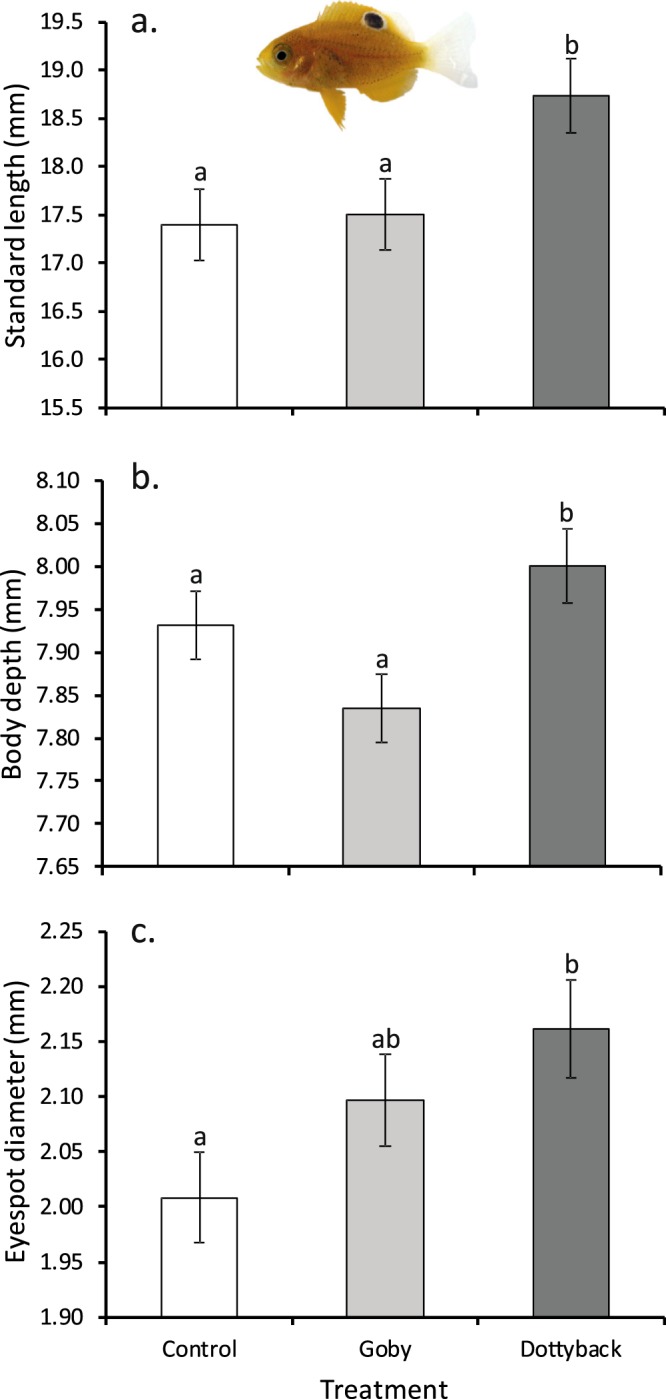
Comparison of (**a**) standard length, (**b**) body depth and (**c**) false eyespot diameter (mean ± SE) of juvenile *Pomacentrus amboinensis* exposed to an empty control, an omnivorous goby, or a predatory dottyback for 45 days. Data are averaged over water source as this had no effect on the variables. Values for body depth and eyespot diameter were adjusted for their covariation with standard length. Letters above bars represent post-hoc groupings of means. N = 47, 45, 41 (left to right). Photographic credit M. McCormick.

There was a significant difference in the size of damselfish at the end of the experiment among treatments (F_2,126_ = 3.77, P = 0.026, η_p_^2^ = 0.06), though no difference between water sources, and no interaction between treatment and water source (P > 0.05). Fish were significantly longer when exposed to the predatory dottybacks, compared to when exposed to the goby or the blank controls (Fig. 1a). Body weight at the end of the experiment showed exactly the same patterns of significance among treatments as standard length (F_2,127_ = 3.86, P = 0.02, η_p_^2^ = 0.06). There was also a similar pattern in the body depth with the effect of standard length removed. Body depth of experimental *P. amboinensis* differed among treatments (F_2,126_ = 4.07, P = 0.019, η_p_^2^ = 0.06; Fig. 1b), though not between water sources, and there was no interaction between treatment and water source (P > 0.05).

**Figure 2:**
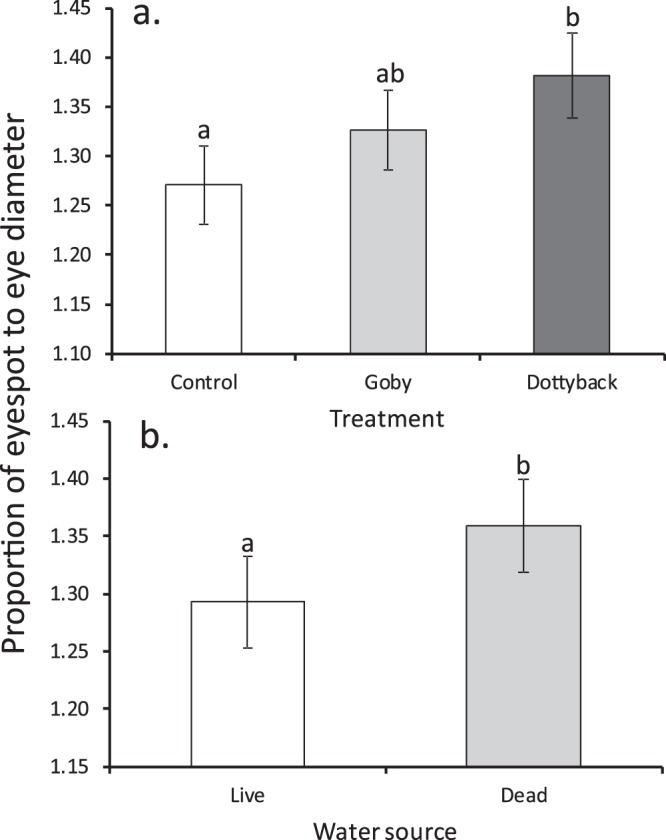
Comparison of proportion of the false eyespot to eye diameter of juvenile *Pomacentrus amboinensis* (mean ± SE) exposed for 45 days to: (**a**) three treatment levels of adult fish (blank control, an omnivorous goby, or a predatory dottyback), and (**b**) two water sources (live coral or dead-degraded coral). Letters above bars represent Fisher’s LSD groupings of means. N = 47, 45, 41 (top); 66, 67 (bottom).

The maximum diameter of the eye was larger for damselfish reared in live coral water compared to those from dead coral water at the end of the experiment (F_1,128_ = 8.33, P = 0.005, η_p_^2^ = 0.06; mean ± SE, Live 1.60 ± 0.01 mm, n = 66; Dead 1.56 ± 0.01 mm, n = 67), but there was no treatment effect or interaction (P > 0.3). The false eyespot diameter differed only among treatments (F_2,127_ = 3.23, P = 0.04, η_p_^2^ = 0.05, with SL as a significant covariate; Fig. 1c), with those damselfish exposed to dottybacks possessing larger eyespots compared to controls. There was a difference in the proportion of the eyespot to eye maximum diameter among treatments (F_2,127_ = 4.0, P = 0.02, η_p_^2^ = 0.06; Fig. 2a) and by water source (F_1,127_ = 4.31, P = 0.04, η_p_^2^ = 0.03; Fig. 2b), but there was no interaction between factors (P = 0.25). False eyespots were proportionately larger in fish exposed to dottybacks, and in those reared in degraded water (Fig. 2b). The relationship between body weight and standard length (i.e., length standardized weight) differed at the level of the interaction between treatment and water source (F_2,121_ = 3.37, P = 0.04). Further analyses showed that this difference was caused by fish exposed to live-coral water and dottybacks being different in slope and intercept than all other relationships (ANCOVA Interaction F_1,129_ = 20.5, P < 0.0001), which did not differ from one another. *P. amboinensis* exposed live coral with dottybacks gained weight at a rate of 42.9 mg/mm, compared to 34.7 mg/mm of all the other treatment combinations combined.

### Behaviour

**Figure 3:**
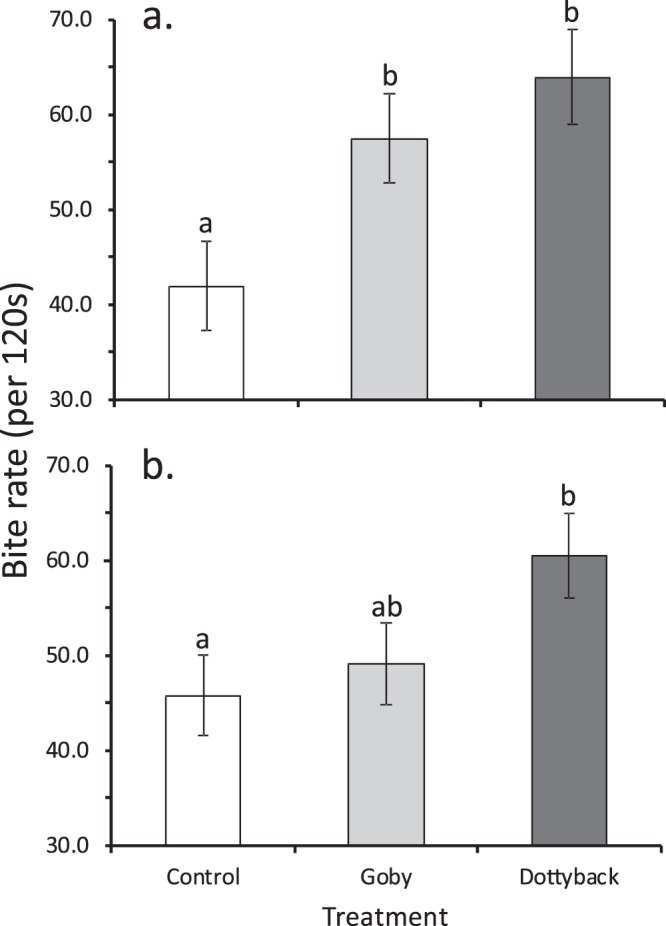
Bite rate (per 120 s; mean ± SE) of juvenile *Pomacentrus amboinensis* (**a**) 4 days after the start of the experiment and (**b**) at the end (day 45) of the experiment. Juvenile damselfish were exposed to three treatment levels: blank control, an omnivorous goby, or a predatory dottyback. Letters above bars represent Fisher’s LSD groupings of means. N = 46, 45, 41 (top), 47, 45, 41 (bottom).

Feeding rate on the fourth day after the initiation of the experiment differed among treatments (F_2,126_ = 5.61, P = 0.005, η_p_^2^ = 0.08), but not by water source or their interaction (P > 0.2). *P. amboinensis* exposed to the goby or dottyback had one-third greater feeding rates than fish within the control treatment (Fig. 3). By the end of the experiment, the lack of effect of water source was still evident (P > 0.3), but the pattern among treatments had changed to a trend in which fish in the dottyback treatment eat more than the control fish (F_2,127_ = 3.07, P = 0.05, η_p_^2^ = 0.05), while goby-exposed juveniles had an intermediate feeding rate.

### Routine swimming and escape performance

The total distance moved by damselfish during routine swimming in the performance arena was affected by water source (F_1,123_ = 5.93, P = 0.01, η_p_^2^ = 0.05), but not by treatment or their interaction (P > 0.7). Fish that had been reared in water that had flowed over dead-degraded coral moved 13% further during their routine swimming compared to those that had been reared in water flowed over live coral (5.1 ± 0.2 vs 4.4 ± 0.2 m/120 s).

There was a positive relationship between distance of the focal fish to startle stimulus and response latency, but this differed significantly among treatments (F_2,119_ = 4.12, P = 0.019, η_p_^2^ = 0.06), with the relationship being much weaker and shallower for fish exposed to dottybacks (r^2^ = 0.07, n = 40) compared to the other two treatments (control r^2^ = 0.37, n = 47; goby r^2^ = 0.47, n = 41).

**Figure 4:**
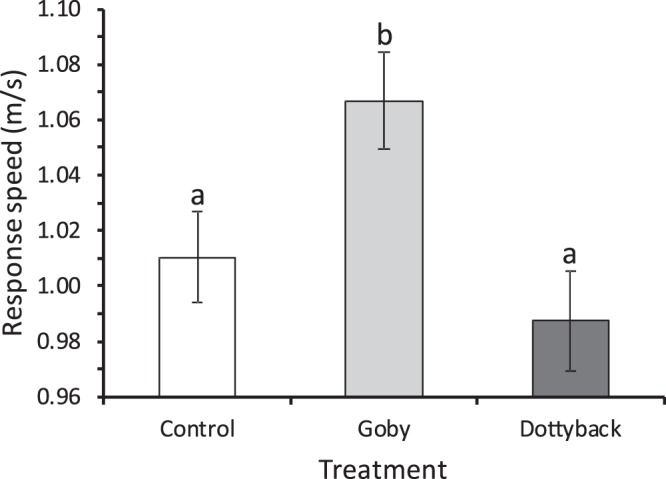
Response speed (mean ± SE) of juvenile *Pomacentrus amboinensis* to a drop stimulus when reared under three treatment levels of adult fish: a blank control, an omnivorous goby, or a predatory dottyback. Values are corrected for their covariation with standard length. Letters above bars represent Fisher’s LSD groupings of means. N = 41, 47, 40.

Average and maximum response speed were positively correlated with length, so SL was used as a significant covariate in the analyses of these variables. Average response speed was affected by treatment (F_2,121_ = 5.38, P = 0.006, η_p_^2^ = 0.08; Fig. 4), but not by water source or their interaction (P > 0.14). Response speed was greater for fish with a history of exposure to gobies than either dottyback or nil controls (Fig. 4). The relationship between maximum speed and standard length differed among treatments (F_2,119_ = 3.79, P = 0.02, η_p_^2^ = 0.06), and once corrected for the effect of the covariate there was a trend towards an effect of treatment on maximum speed (F_2,119_ = 3.17, P = 0.046, η_p_^2^ = 0.05), with those fish exposed to gobies having higher maximum speeds compared to the other two treatments.

## Discussion

The widespread and increasing degradation of coral reefs has drawn attention to the importance of identifying the mechanisms that make one species more resilient to habitat change than others^41,42^, as it has for other ecosystems such as forests^43^. This requires detailed information about the interactions between individuals within the context of their environment, and the potential for phenotypic plasticity to aid in their adaption to new environmental conditions. The current experiment is one of the first studies to explore how the changing nature of an ecosystem may affect predator-induced trait-mediated effects on body shape, behaviour and performance. Our findings show that prey attributes were flexible to predator presence and environmental context, but the overall effect of degraded water on trait-mediated predator effects was weaker than predicted. While there was a relatively minor impact of habitat degradation compared to the effect of predator cues, these modifications to the physiology of early settlement-stage individuals may be important in influencing life-history and survival trajectories at this important life-history bottleneck^44,45^ and on subsequent life stages^46^.

Water from degraded habitat did affect some aspects of the morphology and behaviour of fish, but in general this effect was independent of (i.e., did not interact with) whether fish were exposed to predators or not. The maximum diameter of the eye was larger for fish from live coral water compared to those reared in dead coral water. Since smaller eyes tend to have lower visual acuity^47^, smaller eyes may represent a cost to fish in degraded water^48^. These findings meant that damselfish reared in dead-degraded coral water had larger ocellus sizes relative to their eyes compared to fish reared in water from live coral. Interestingly, the effect of degraded water on ocellus size had half the effect size of the effect of predators on relative ocellus size, which caused predator-exposed fish to have larger ocellus relative to the true eye in keeping with a previous study^23^. Having a proportionally larger false eyespot compared to the true eye may be an advantage as it may attract strikes to the tail rather than the vulnerable head region, as has been shown by birds preying on butterflies with wing eyespots^49^. Predators anticipate the direction prey will move after an attack is initiated, and an eyespot may lead predators to misjudge the prey’s centre of mass^50^. A prey attacked at the caudal area can also escape and survive^51^, however an attack on the head would damage vital parts reducing the likelihood of survival. McPhail (1977) demonstrated that caudal spots in a freshwater characid fish (*Hyphessobrycon panamensis*) deflect the aim of a predatory freshwater barracuda (*Ctenolucius beani*). McPhail found that more predators focused their attacks on the caudal area of prey fish that had an artificial caudal spot drawn on compared to fish with no spot. Prey with these artificial eyespots escaped predators more frequently than fish with no eyespots, demonstrating a clear advantage. The presence of strongly selective predation^52^, and the phenotypic plasticity displayed by juvenile fishes generally (e.g.^53,54^), suggests that the benefits of developing a larger relative false eyespot outweighs the costs of having a smaller true eye, though field test will be needed to support this assertion.

Fish reared in degraded coral water moved 13% more during routine activity assessments compared to fish from healthy coral water. Previous research suggests that whether greater movement is advantageous depends on the life stage of the individual and knowledge of the specific risks associated with the settlement site^55^. Marine organisms with complex life histories that settle after a pelagic larval phase, be they invertebrates or vertebrates like fishes, often lack information on the identity of predators in their specific settlement site^56^. Research on fishes suggest that they rapidly obtain information on local risks through their own interactions with the community (e.g.^39^) and through the publically available information^57^. One study that quantified the space use of newly settled fish in the field found that survival on habitat patches was higher for bolder, more active fish, but was negatively related to how far they were willing to stray from habitat^55^. Previous research examining the behaviour of fishes on habitat patches found that fish on dead-degraded coral are more active and stay further away from the potential shelter^31,58^. The inability of many fishes to use alarm odours to assess and learn about risk may mean that fish on dead coral may be less conservative in their space use because they are unable to judge risk effectively. The problem they face is the impossibility of knowing their risk judgement has been impaired because, unlike senses such as vision, they have no way of knowing that they are not detecting an important odour. Such risky behaviour has been shown to lead to 75% higher mortality rates of juvenile fish over the first 48 h after settlement^31^ and may lead fish on degraded habitats to have higher mortality (e.g.^33,59^).

Regardless of the effects of whether the nursery habitat was live or dead-degraded coral, our findings show strong effects of the presence of predators on body shape, pigmentation, behaviour and performance in line with the limited studies that have explored these trait-mediated predator effects on fishes^23,24^. Studies of other organisms have found that non-consumptive predator-induced morphological change occurs in diverse taxa including amphibian^60^, gastropods^61^ and ciliates^62^. In the present experiments, fish exposed to predators grew longer compared to fish exposed to omnivorous gobies or the blank control. Being larger than average at a specific developmental stage is commonly advantageous due to all the aspect of performance that correlate with length^63,64^. The current experiment found aspects of performance such routine activity, maximum and mean escape speed scaled positively with length, emphasizing the importance of size. Many studies have found that predators select for the smaller members of a prey group^65^, and numerous studies within the current system demonstrate a strong and ongoing advantage to being larger than average in the early juvenile stage^66,67^.

Our study also found that body depth was also promoted by exposure to predators, in support of previous studies. The gape-limited nature of many aquatic predators means that one of the key relationships underlying catch success may be the width of a predator’s mouth compared to prey body depth^67–69^. Our finding of the development of a deeper body when in close proximity with a predator matches the findings of Lönnstedt *et al*.’s (2013) study of the same species, and the response of the crucian carp to predatory pike cues^24^. In a laboratory experiment, Chivers, *et al*.^25^ found that the relative body depth of the crucian carp was dynamic and could be rapidly altered though exposure to olfactory cues. This and the current experiment emphasize the dynamic nature of morphological defenses to the non-consumptive effects of predators.

Being exposed to a predator affected aspects of body length and growth compared to blank controls. Having the smell and sight of a known predator affected growth in length and weight, but there was an interaction between water source and treatment when length-standardised weight (i.e., bulk) was considered. Fish exposed to predators in live coral water gained more body mass than other treatment combinations. Given that all fish were provided the same amount of food throughout the experiment, this suggest that the efficiency of food conversion to tissue may have been higher for predator-exposed fish in live coral water compared to those in water from dead-degraded coral. Although the mechanism that would support this is unclear, it is likely related to the stress hormone cortisol. Middlemis *et al*.^70^ found that tadpoles (*Rana sylvatica*) that were exposed to predators had higher cortisol, which directly induced substantial growth of the tail compared to controls not exposed to predators. Our findings contrast with a number of other studies that have examined the influence of predator presence on fish growth^71^. For instance, Fu *et al*.^72^ exposed juvenile Chinese bream (*Parabramis pekinensis*) prey to predatory southern catfish (*Silurus meridionalis*) and found reduced growth and higher metabolism in prey groups with predators. Differences in results among studies may be due to differences in the life stage of fish examined, with other studies examining late-stage juveniles. The current study was initiated when individuals were newly metamorphosed; a life stage previously shown to be highly flexible in morphology and behaviour (e.g.^22,23,55^). Clearly, the physiological mechanisms underlying this flexibility and how it was affected by water from degraded corals warrant further research.

Fish reared with predators tended to have a uniformly fast escape response latency, which differed compared to the other treatments, where there was a strong relationship between distance to the startle stimulus and latency. This shows fish that had been exposed to predators were more responsive and responded more quickly. Fish have recently been shown to exhibit a context-dependent escape response, such that they optimize when and how fast they respond to a startle stimulus (such as a predator strike) dependent on how highly they rank the threat^40,73,74^. It appears that predator-exposed fish are primed to burst regardless of the distance to the threat, while fish reared under more benign conditions had an optimized response. Interestingly, once the fast-start had been initiated the goby-reared fish had better performance once they had initiated a C-start. Given that the speed at which fish respond to a predator (i.e., latency) is one of the best predictors of survival in the field^64^, it appears likely that the priming that comes with being forewarned of the presence of a predator may lead to higher survival.

Given the findings of previous research on the influence of degraded water on the efficacy of alarm odours to label threats and assess risk, it was surprising that the present study found few interactions between water source and predator presence. Laboratory and field studies show that our focal species, like many fishes^29^, is unable to use chemical alarm odours to learn predator identity or update information in the presence of small amounts of water from dead-degraded coral^32^, or even on a live coral patch surrounded by degraded habitat^33^. Being on thermally bleached or dead corals also changes the space use, activity and interactions of fishes^59^. Given the ability of cues from degraded coral to modify the availability of cues and behaviour it was expected that such water would also have a major impact on trait-mediated predator effects. While the damselfish may not have initially recognized the dottybacks as predators, it is likely that the close proximity of the predator to prey, and the strikes of predators at the Perspex dividers may have elevated the ranking of dottybacks on the threat gradient^75^. Other non-olfactory learning mechanisms, such as visual strikes, have likely filled the information void that would usually be received from olfactory sources^76^. While research suggests that some form of sensory compensation may occur when visual cues are reduced (through turbulence or in darkness^77^;), this has not been demonstrated for olfactory cues because of the uncertain meaning of an absence of smell. Clearly however, the present study demonstrates that there is substantial compensation for documented loss of the olfactory threat information in water from dead-degraded habitats^27^. This information allows the trait-mediated predator effects to be fully demonstrated in degraded water. It remains to be determined how they achieve the compensation for the loss of the important associative-learning mechanism of predator learning and reinforcement, and when the compensation occurs to allow the development of trait-mediated predator effects. Understanding these mechanisms will be key to determining how species survive under environmental adversity and how species’ resilience is promoted.

## Supplementary information

Details on the study system, animal welfare, tank setup and morphometrics

## Data Availability

Data is accessible from the Tropical Data Hub (10.25903/5cb82367e7f39).
