# Supplementary material for: Habitat degradation and predators have independent trait-mediated effects on prey
Source: Sci Rep. 2019 Oct 31;9:15705. doi: 10.1038/s41598-019-51798-2 (PMC6823502; doi:10.1038/s41598-019-51798-2)
Supplement: Supplementary file 1 — Details on the study system, animal welfare, tank setup and morphometrics [file 41598_2019_51798_MOESM1_ESM.pdf]

Supplementary file

McCormick MI, Fakan EP, Palacios MM. Habitat degradation and predators have independent trait-mediated effects on prey

Study system

The focal damselfish species, *Pomacentrus amboinensis*, is a common component of Indo-Pacific coral reefs where it is an omnivorous planktivore [1]. The species settles after a pelagic larval phase of 15–23 days and 10-15 mm standard length (SL). Their metamorphosis occurs at night and involves a rapid (2-6 h) change in colouration with the development of a false eyespot (ocellus) on the posterior dorsal fin (Supplementary Fig. S2), which is kept until maturation at ~ 40 mm SL [2]. Light traps catch fishes principally at the end of their larval phase and they are largely naïve to the identity of reef-based predators.

Animal welfare details

Research was conducted in accordance with the James Cook University Animal Ethics guidelines with approval from the JCU Animal Ethics Committee (approval A2408). Transportation of juvenile fishes from light traps to the Lizard Island research station aquarium system took 10 to 20 minutes and occurred in 60 L tanks with lids to reduce stress by subduing the ambient light conditions. The station aquarium system pumps water straight from the lagoon and has similar chemistry and temperature to ambient seawater. Target fishes were carefully removed from the catch with hand nets, while the bicatch was returned to the reef after being temporarily maintained (< four h) in a flow-through seawater system. *P. amboinensis* were kept in 35 L aquaria of aerated seawater and fed newly hatched *Artemia* spp. nauplii three-times a day to satiation. Fish were kept for 48 h prior to being used in the experiment to make sure they had recovered from the stress associated with capture. During the experiment, juvenile damselfish (~ 11-15 mm SL) that were fed to the *Ps. fuscus* were killed by cold-shock. This involved placing fishes in a slurry of ice and aerated seawater

(one:five mix) and leaving them for > five min. The ice floated on the top of the container and almost immediately (< 30 s) fish sank to the bottom, ventilated slowly and irregularly and lost reflex reactivity (i.e., reached stage five sedation, [3]) followed quickly by a cessation of ventilation (stage six). Euthanasia by cold shock has been shown to be more reliable and cause less distress than anesthesia overdose [4].

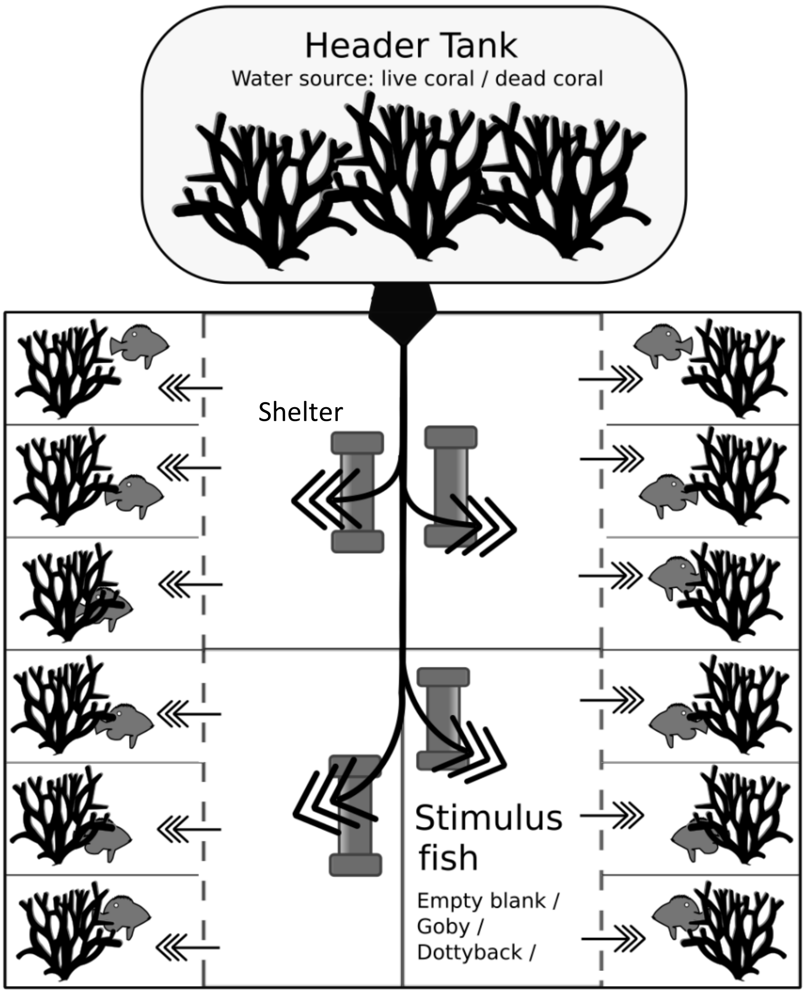

Figure S1. Schematic diagram of the experimental setup. Water from the lagoon flowed through a 1 micron filter into a 60 litre header tank containing live *Pocillopora damicornis* hard coral or dead-degraded coral (Water source). The water then entered the 6.1 litre tank compartment (32.1 x 10.5 x 18 cm) that housed the adult stimulus fishes with shelter (Treatment: no fish, a goby *Amblygobius phalaena*, or a dottyback predator *Pseudochromis fuscus*), and passed via holes in the Perspex wall into the separate 2.3 litre tanks (10.7 x 12 x 18 cm) containing the focal juvenile damselfish *Pomacentrus amboinensis*. Water exited through holes in the back of the 12 individual compartments. Tanks were overall 52 litres in size (64.2 x 45 x 18 cm). PVC tubes providing shelter to the stimulus species (7 cm long x 4

cm dia.) and the prey (3 cm long x 4 cm dia.). The complete experimental setup was replicated 12 times.

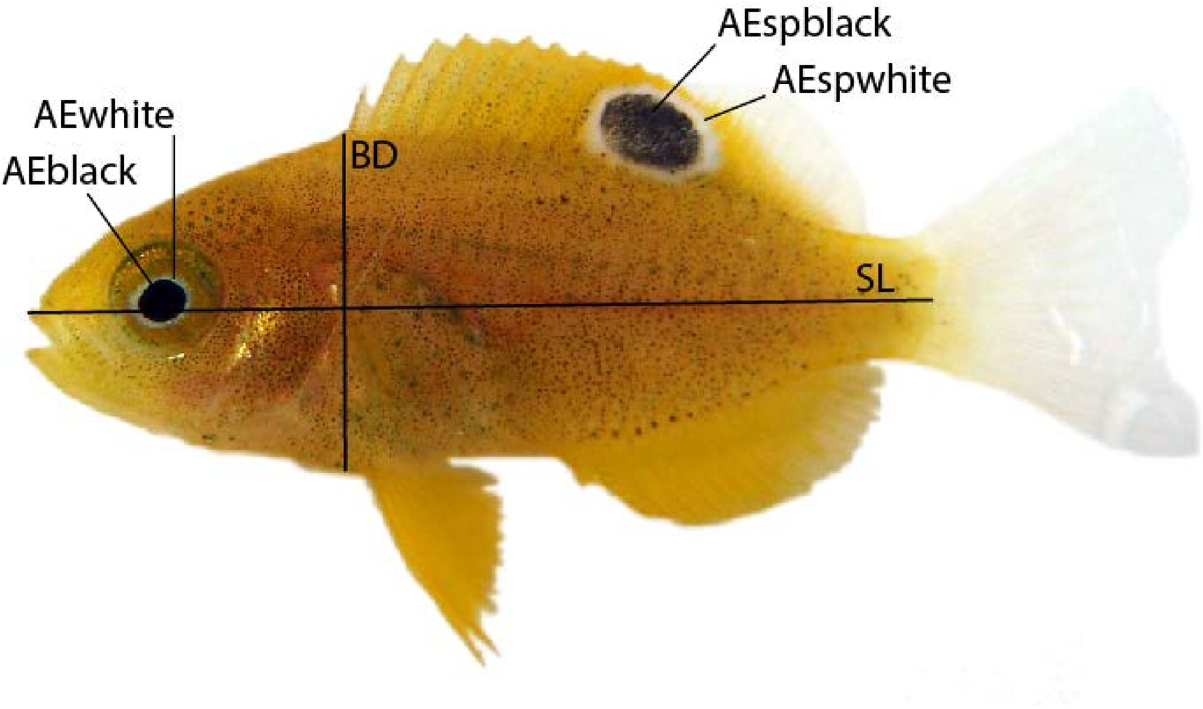

Figure S2. Morphological characteristics measured to examine the impact of water source and predator effects on juvenile *Pomacentrus amboinensis*. SL – standard length; BD – body depth; AEwhite – area of the eye white; AEblack – area of eye black; AEspwhite – area of the eyespot white; AEspblack – area of eyespot black. Photographic credit: M. McCormick

References

1 McCormick, M. I. & Weaver, C. J. It pays to be pushy: intracohort interference competition between two reef fishes. *PloS One* **7**, e42590 (2012).

2 McCormick, M. I. Protogyny in a tropical damselfish: females queue for future benefits. *PeerJ* **4**, e2198, doi:10.7717/peerj.2198 (2016).

3 Javahery, S., Nekoubin, H. & Moradlu, A. H. Effect of anaesthesia with clove oil in fish (review). *Fish Physiol. Biochem.* **38**, 1545-1552, doi:10.1007/s10695-012-9682-5 (2012).

4 Wilson, J. M., Bunte, R. M. & Carty, A. J. Evaluation of rapid cooling and tricaine methanesulfonate (MS222) as methods of euthanasia in zebrafish (*Danio rerio*). *Journal of the American Association for Laboratory Animal Science* **48**, 785-789 (2009).
